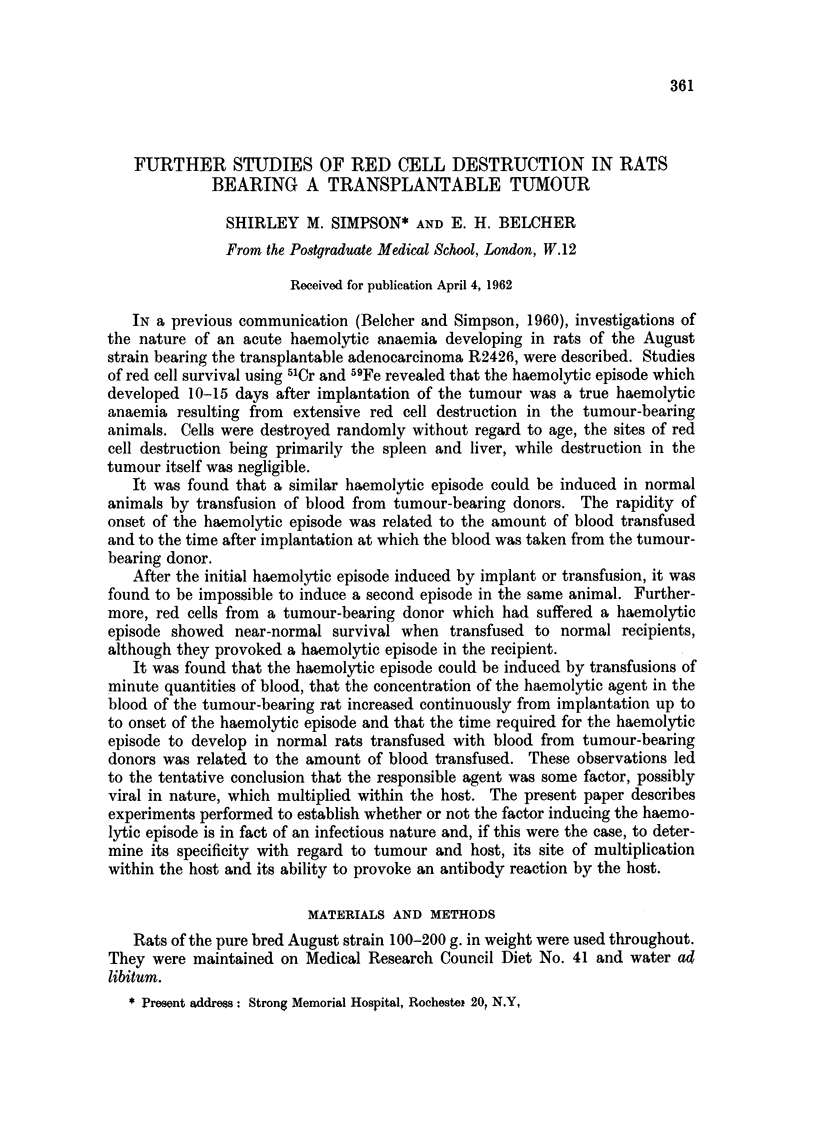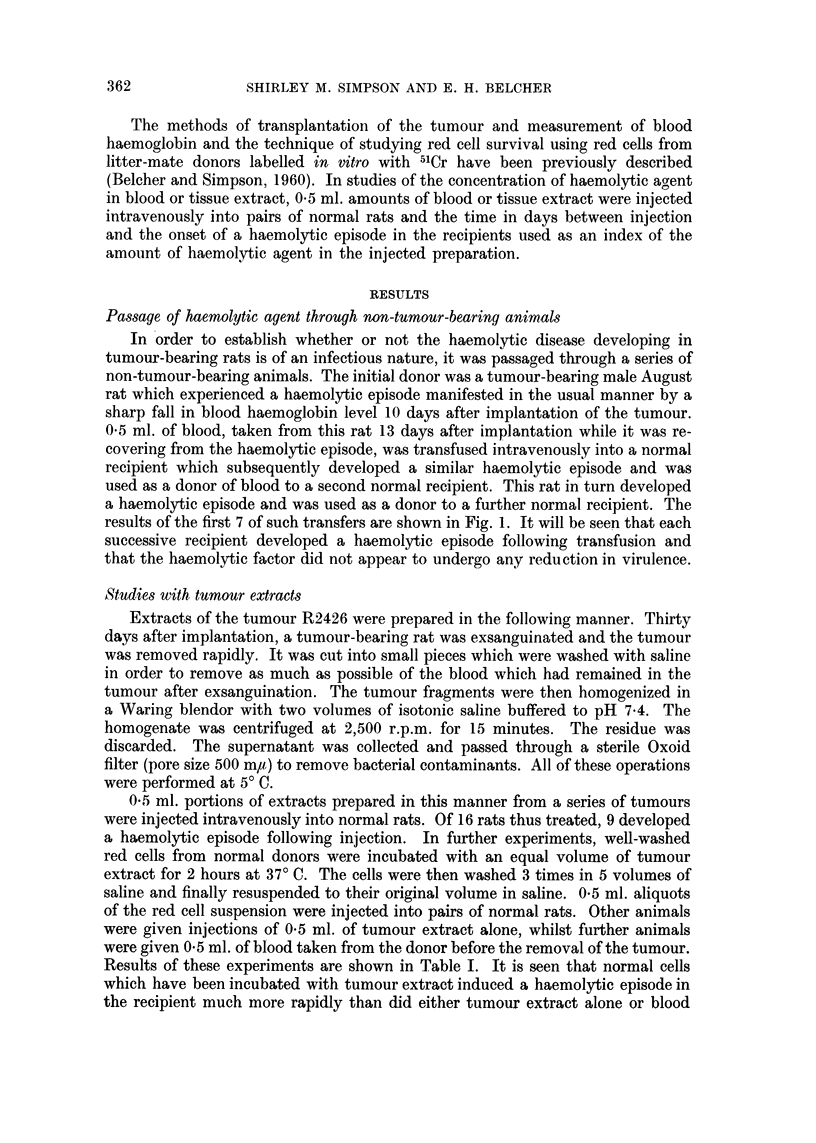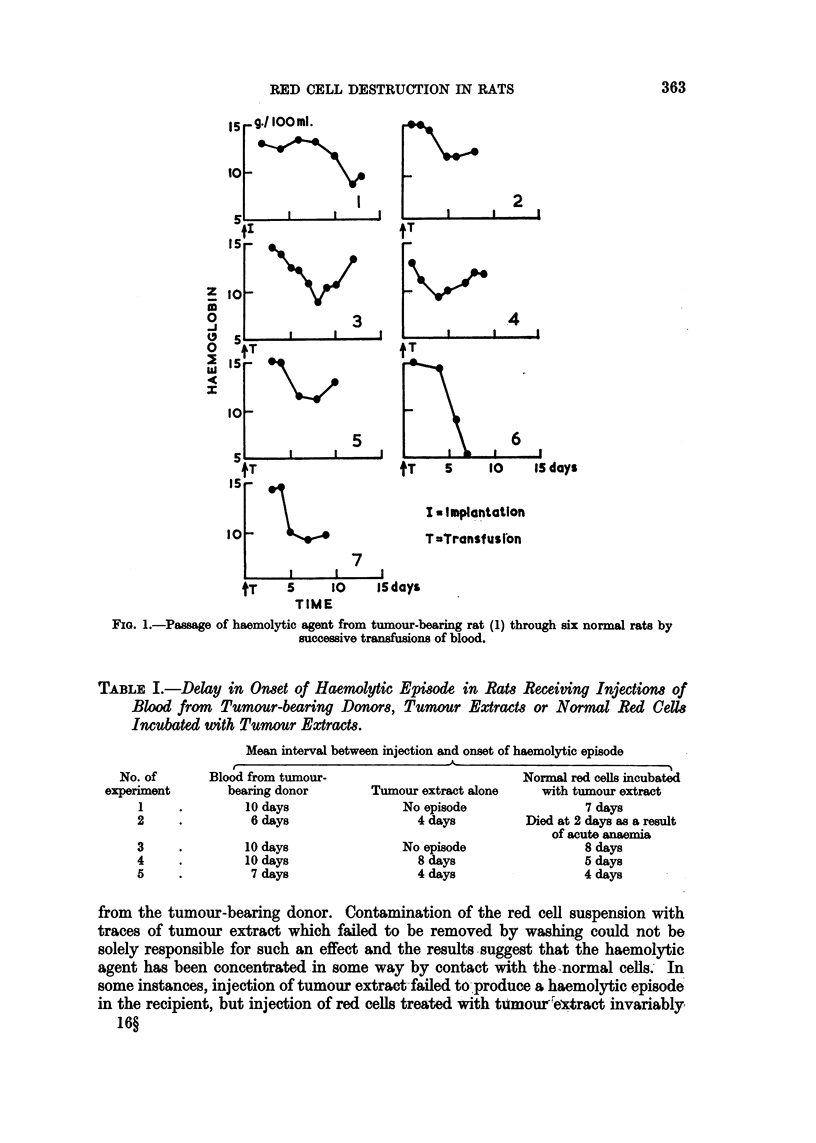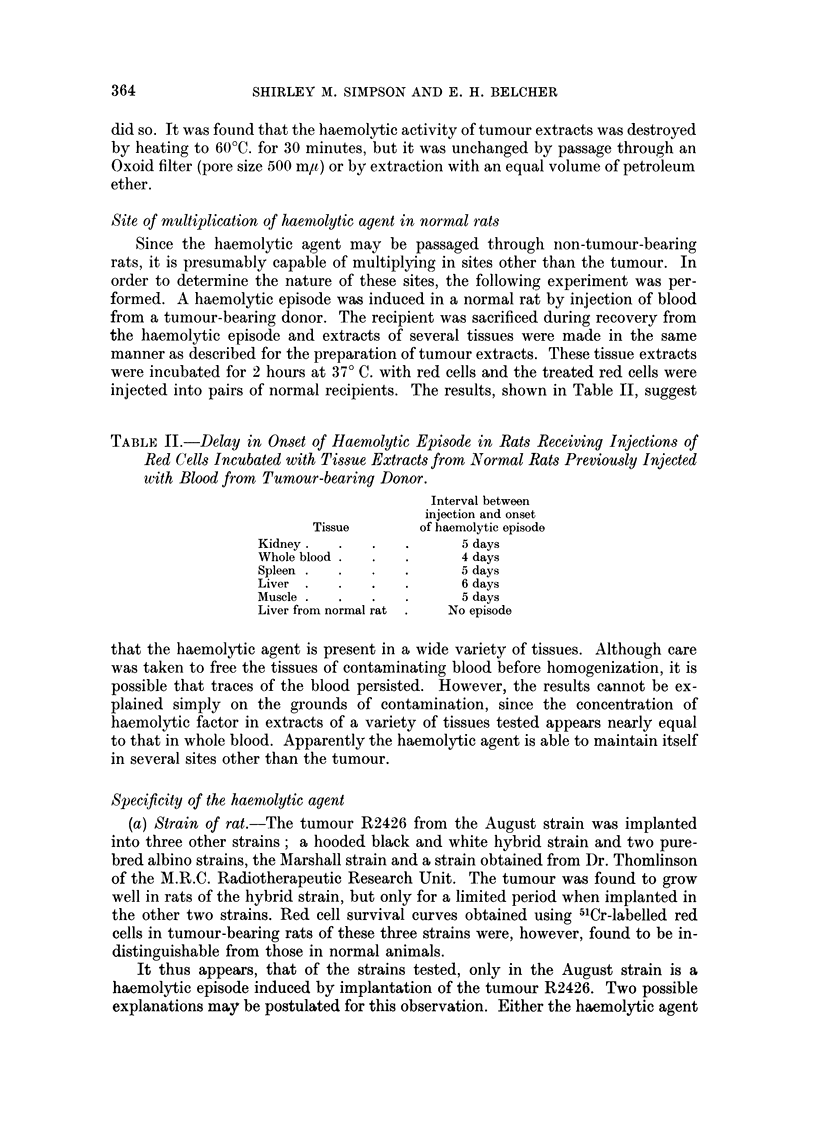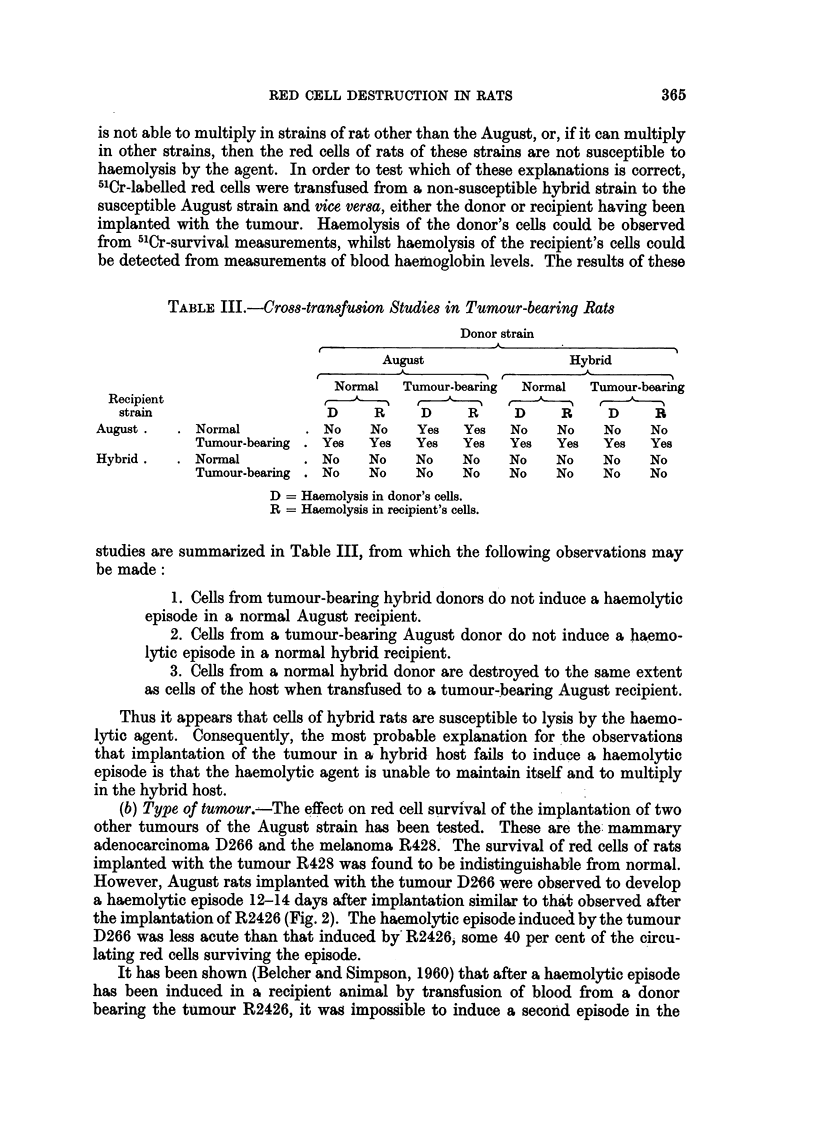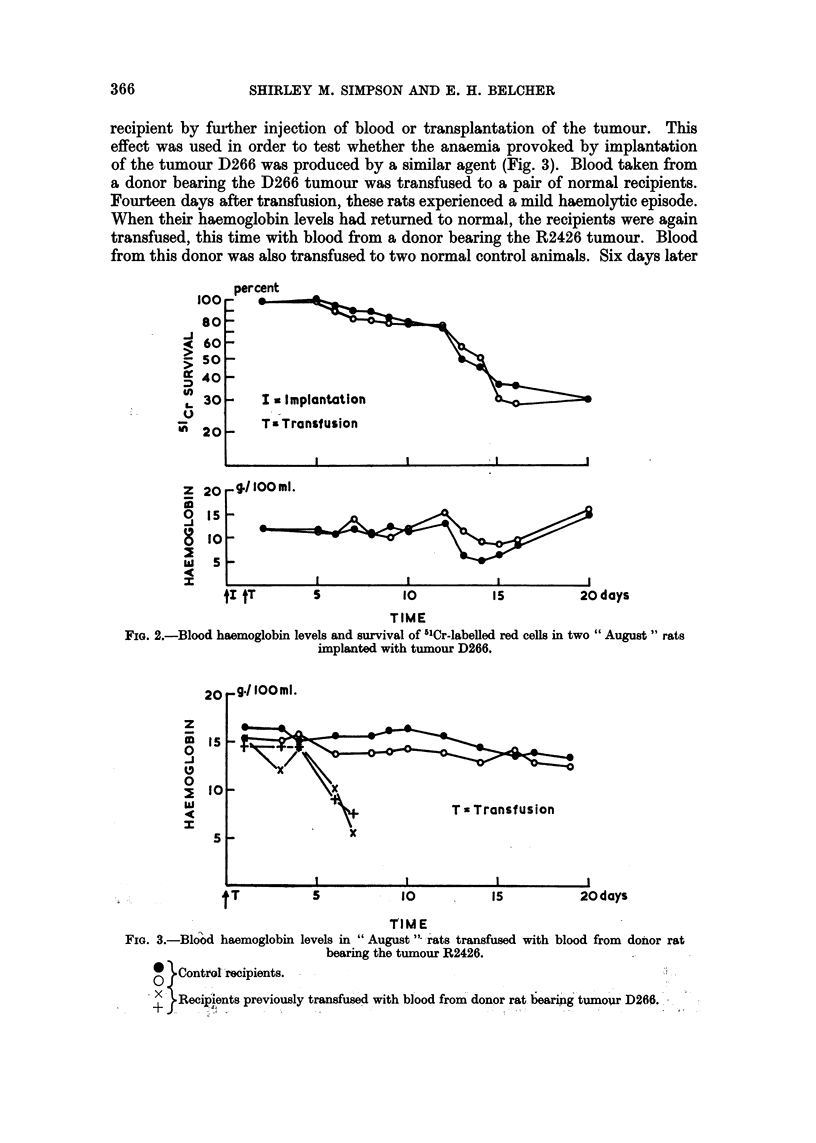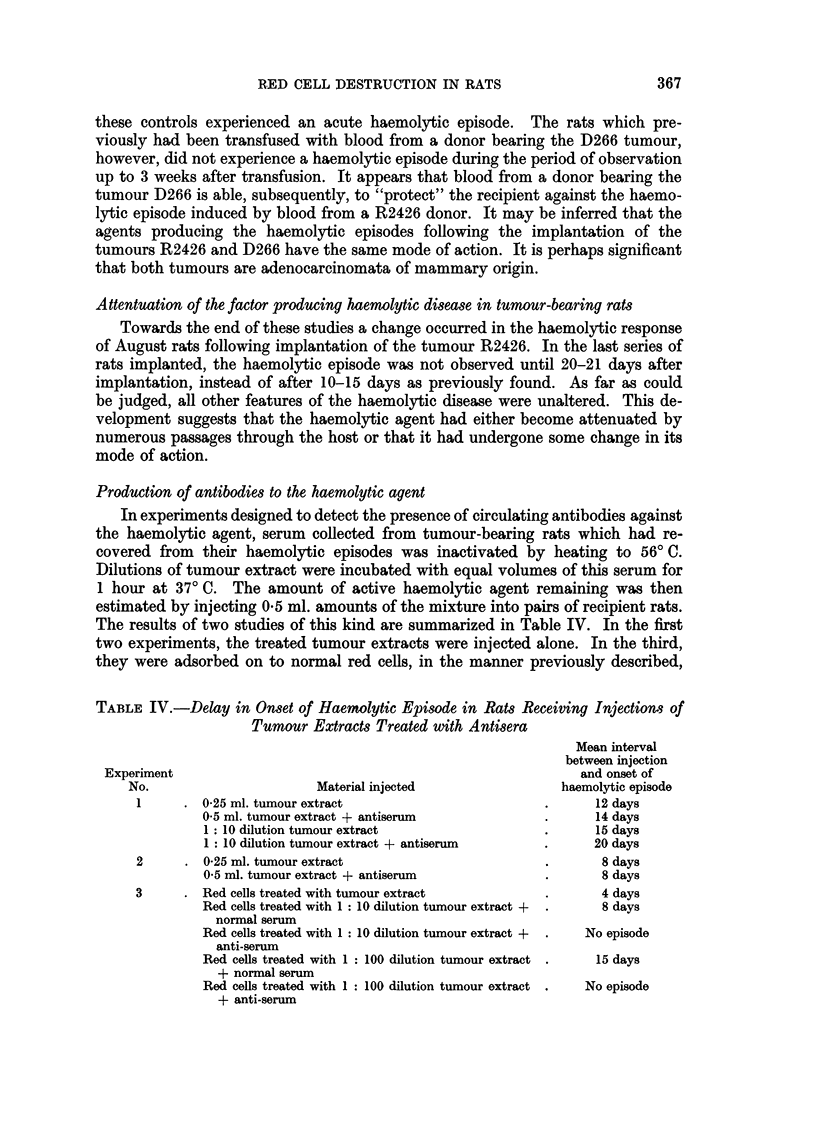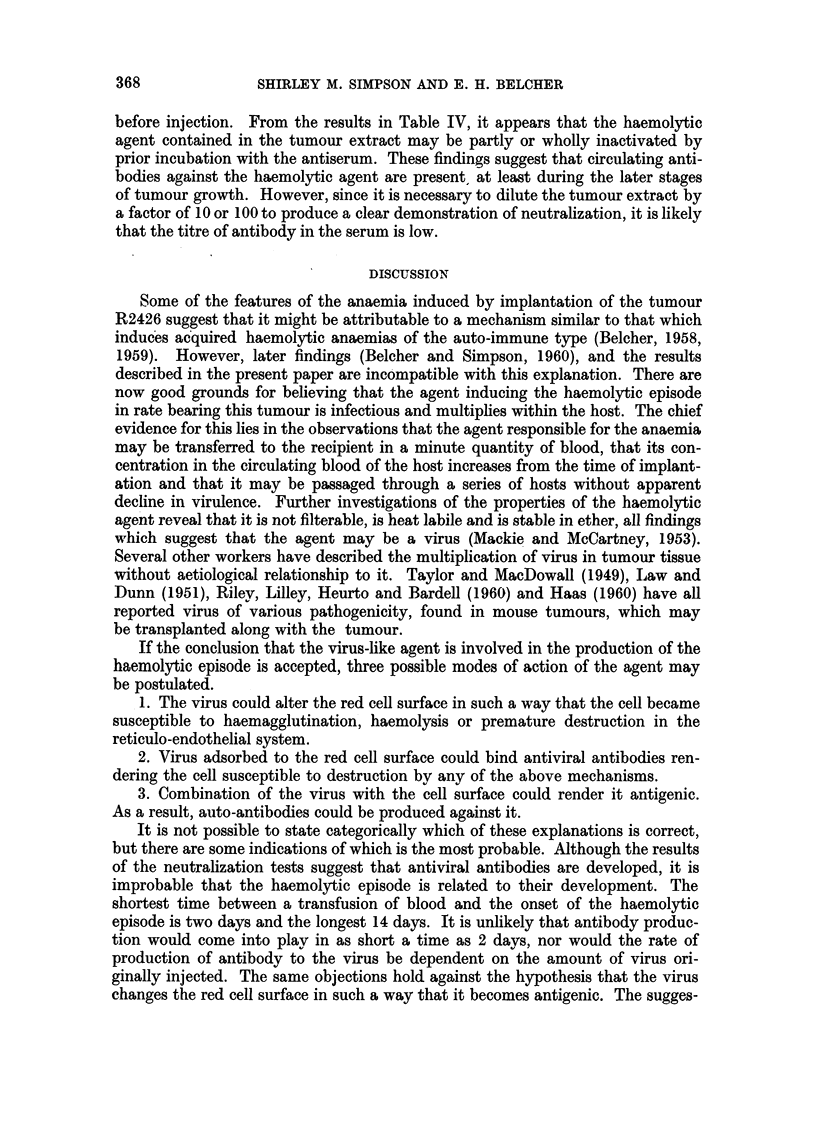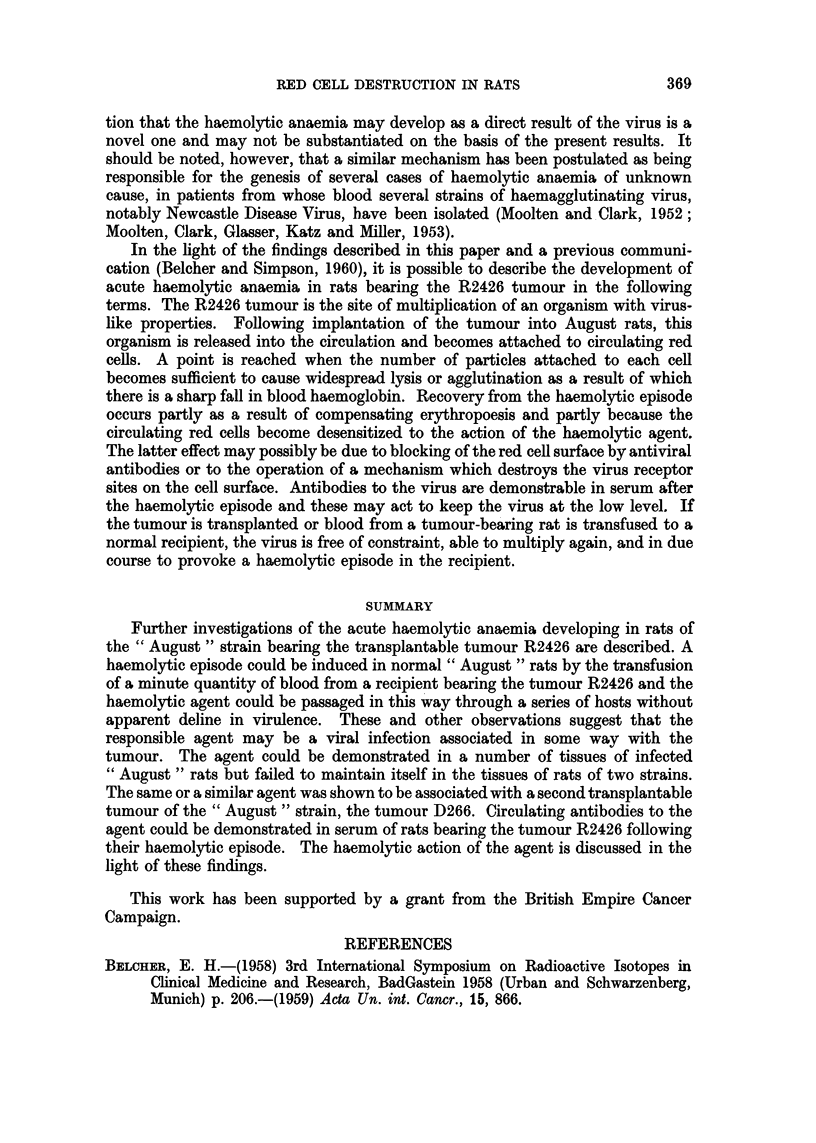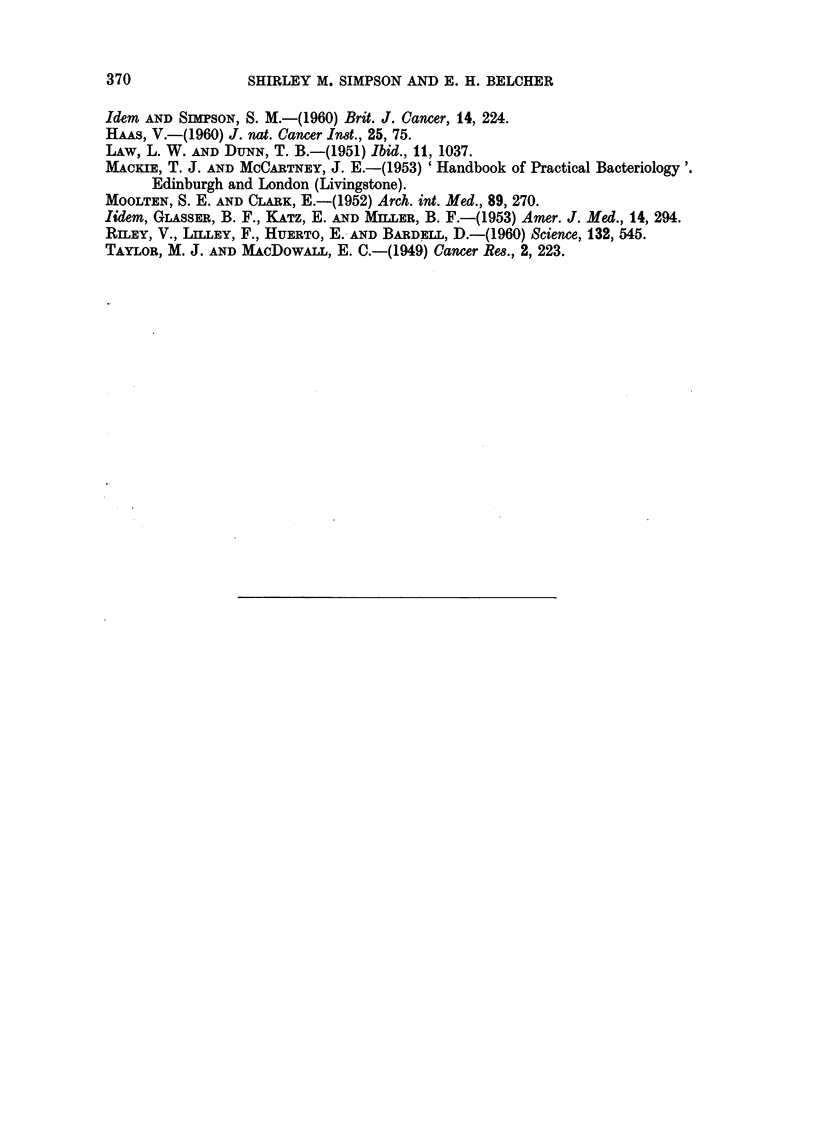# Further Studies of Red Cell Destruction in Rats Bearing a Transplantable Tumour

**DOI:** 10.1038/bjc.1962.41

**Published:** 1962-06

**Authors:** Shirley M. Simpson, E. H. Belcher


### FURTHER STUDIES OF RED CELL DESTRUCTION IN RATS BEARING A TRANSPLANTABLE TUMOUR

SHIRLEY M. SIMPSON* AND E. H. BELCHER

*From the Postgraduate Medical School, London, W.12*

Received for publication April 4, 1962

IN a previous communication (Belcher and Simpson, 1960), investigations of the nature of an acute haemolytic anaemia developing in rats of the August strain bearing the transplantable adenocarcinoma R2426, were described. Studies of red cell survival using $^{51}Cr$ and $^{59}Fe$ revealed that the haemolytic episode which developed 10–15 days after implantation of the tumour was a true haemolytic anaemia resulting from extensive red cell destruction in the tumour-bearing animals. Cells were destroyed randomly without regard to age, the sites of red cell destruction being primarily the spleen and liver, while destruction in the tumour itself was negligible.

It was found that a similar haemolytic episode could be induced in normal animals by transfusion of blood from tumour-bearing donors. The rapidity of onset of the haemolytic episode was related to the amount of blood transfused and to the time after implantation at which the blood was taken from the tumour-bearing donor.

After the initial haemolytic episode induced by implant or transfusion, it was found to be impossible to induce a second episode in the same animal. Furthermore, red cells from a tumour-bearing donor which had suffered a haemolytic episode showed near-normal survival when transfused to normal recipients, although they provoked a haemolytic episode in the recipient.

It was found that the haemolytic episode could be induced by transfusions of minute quantities of blood, that the concentration of the haemolytic agent in the blood of the tumour-bearing rat increased continuously from implantation up to to onset of the haemolytic episode and that the time required for the haemolytic episode to develop in normal rats transfused with blood from tumour-bearing donors was related to the amount of blood transfused. These observations led to the tentative conclusion that the responsible agent was some factor, possibly viral in nature, which multiplied within the host. The present paper describes experiments performed to establish whether or not the factor inducing the haemolytic episode is in fact of an infectious nature and, if this were the case, to determine its specificity with regard to tumour and host, its site of multiplication within the host and its ability to provoke an antibody reaction by the host.

### MATERIALS AND METHODS

Rats of the pure bred August strain 100–200 g. in weight were used throughout. They were maintained on Medical Research Council Diet No. 41 and water *ad libitum.*

* Present address: Strong Memorial Hospital, Rochester 20, N.Y,

The methods of transplantation of the tumour and measurement of blood haemoglobin and the technique of studying red cell survival using red cells from litter-mate donors labelled *in vitro* with $^{51}$Cr have been previously described (Belcher and Simpson, 1960). In studies of the concentration of haemolytic agent in blood or tissue extract, 0·5 ml. amounts of blood or tissue extract were injected intravenously into pairs of normal rats and the time in days between injection and the onset of a haemolytic episode in the recipients used as an index of the amount of haemolytic agent in the injected preparation.

### RESULTS

### *Passage of haemolytic agent through non-tumour-bearing animals*

In order to establish whether or not the haemolytic disease developing in tumour-bearing rats is of an infectious nature, it was passaged through a series of non-tumour-bearing animals. The initial donor was a tumour-bearing male August rat which experienced a haemolytic episode manifested in the usual manner by a sharp fall in blood haemoglobin level 10 days after implantation of the tumour. 0·5 ml. of blood, taken from this rat 13 days after implantation while it was recovering from the haemolytic episode, was transfused intravenously into a normal recipient which subsequently developed a similar haemolytic episode and was used as a donor of blood to a second normal recipient. This rat in turn developed a haemolytic episode and was used as a donor to a further normal recipient. The results of the first 7 of such transfers are shown in Fig. 1. It will be seen that each successive recipient developed a haemolytic episode following transfusion and that the haemolytic factor did not appear to undergo any reduction in virulence.

### *Studies with tumour extracts*

Extracts of the tumour R2426 were prepared in the following manner. Thirty days after implantation, a tumour-bearing rat was exsanguinated and the tumour was removed rapidly. It was cut into small pieces which were washed with saline in order to remove as much as possible of the blood which had remained in the tumour after exsanguination. The tumour fragments were then homogenized in a Waring blendor with two volumes of isotonic saline buffered to pH 7·4. The homogenate was centrifuged at 2,500 r.p.m. for 15 minutes. The residue was discarded. The supernatant was collected and passed through a sterile Oxoid filter (pore size 500 m$\mu$) to remove bacterial contaminants. All of these operations were performed at 5° C.

0·5 ml. portions of extracts prepared in this manner from a series of tumours were injected intravenously into normal rats. Of 16 rats thus treated, 9 developed a haemolytic episode following injection. In further experiments, well-washed red cells from normal donors were incubated with an equal volume of tumour extract for 2 hours at 37° C. The cells were then washed 3 times in 5 volumes of saline and finally resuspended to their original volume in saline. 0·5 ml. aliquots of the red cell suspension were injected into pairs of normal rats. Other animals were given injections of 0·5 ml. of tumour extract alone, whilst further animals were given 0·5 ml. of blood taken from the donor before the removal of the tumour. Results of these experiments are shown in Table I. It is seen that normal cells which have been incubated with tumour extract induced a haemolytic episode in the recipient much more rapidly than did either tumour extract alone or blood

FIG. 1.—Passage of haemolytic agent from tumour-bearing rat (1) through six normal rats by successive transfusions of blood.

TABLE I.—*Delay in Onset of Haemolytic Episode in Rats Receiving Injections of Blood from Tumour-bearing Donors, Tumour Extracts or Normal Red Cells Incubated with Tumour Extracts.*

| | Mean interval between injection and onset of haemolytic episode | | |
|---|---|---|---|
| No. of experiment | Blood from tumour-bearing donor | Tumour extract alone | Normal red cells incubated with tumour extract |
| 1 . | 10 days | No episode | 7 days |
| 2 . | 6 days | 4 days | Died at 2 days as a result of acute anaemia |
| 3 . | 10 days | No episode | 8 days |
| 4 . | 10 days | 8 days | 5 days |
| 5 . | 7 days | 4 days | 4 days |

from the tumour-bearing donor. Contamination of the red cell suspension with traces of tumour extract which failed to be removed by washing could not be solely responsible for such an effect and the results suggest that the haemolytic agent has been concentrated in some way by contact with the normal cells. In some instances, injection of tumour extract failed to produce a haemolytic episode in the recipient, but injection of red cells treated with tumour extract invariably

16§

did so. It was found that the haemolytic activity of tumour extracts was destroyed by heating to 60°C. for 30 minutes, but it was unchanged by passage through an Oxoid filter (pore size 500 m$\mu$) or by extraction with an equal volume of petroleum ether.

*Site of multiplication of haemolytic agent in normal rats*

Since the haemolytic agent may be passaged through non-tumour-bearing rats, it is presumably capable of multiplying in sites other than the tumour. In order to determine the nature of these sites, the following experiment was performed. A haemolytic episode was induced in a normal rat by injection of blood from a tumour-bearing donor. The recipient was sacrificed during recovery from the haemolytic episode and extracts of several tissues were made in the same manner as described for the preparation of tumour extracts. These tissue extracts were incubated for 2 hours at 37° C. with red cells and the treated red cells were injected into pairs of normal recipients. The results, shown in Table II, suggest

TABLE II.—*Delay in Onset of Haemolytic Episode in Rats Receiving Injections of Red Cells Incubated with Tissue Extracts from Normal Rats Previously Injected with Blood from Tumour-bearing Donor.*

| Tissue | Interval between injection and onset of haemolytic episode |
|---|---|
| Kidney . . . . | 5 days |
| Whole blood . . . | 4 days |
| Spleen . . . . | 5 days |
| Liver . . . . | 6 days |
| Muscle . . . . | 5 days |
| Liver from normal rat . | No episode |

that the haemolytic agent is present in a wide variety of tissues. Although care was taken to free the tissues of contaminating blood before homogenization, it is possible that traces of the blood persisted. However, the results cannot be explained simply on the grounds of contamination, since the concentration of haemolytic factor in extracts of a variety of tissues tested appears nearly equal to that in whole blood. Apparently the haemolytic agent is able to maintain itself in several sites other than the tumour.

*Specificity of the haemolytic agent*

(*a*) *Strain of rat.*—The tumour R2426 from the August strain was implanted into three other strains ; a hooded black and white hybrid strain and two pure-bred albino strains, the Marshall strain and a strain obtained from Dr. Thomlinson of the M.R.C. Radiotherapeutic Research Unit. The tumour was found to grow well in rats of the hybrid strain, but only for a limited period when implanted in the other two strains. Red cell survival curves obtained using $^{51}$Cr-labelled red cells in tumour-bearing rats of these three strains were, however, found to be indistinguishable from those in normal animals.

It thus appears, that of the strains tested, only in the August strain is a haemolytic episode induced by implantation of the tumour R2426. Two possible explanations may be postulated for this observation. Either the haemolytic agent is not able to multiply in strains of rat other than the August, or, if it can multiply in other strains, then the red cells of rats of these strains are not susceptible to haemolysis by the agent. In order to test which of these explanations is correct, $^{51}Cr$-labelled red cells were transfused from a non-susceptible hybrid strain to the susceptible August strain and *vice versa*, either the donor or recipient having been implanted with the tumour. Haemolysis of the donor's cells could be observed from $^{51}Cr$-survival measurements, whilst haemolysis of the recipient's cells could be detected from measurements of blood haemoglobin levels. The results of these

TABLE III.—*Cross-transfusion Studies in Tumour-bearing Rats*

| | | Donor strain | | | | | | | |
|---|---|---|---|---|---|---|---|---|---|
| | | August | | | | Hybrid | | | |
| | | Normal | | Tumour-bearing | | Normal | | Tumour-bearing | |
| Recipient strain | | D | R | D | R | D | R | D | R |
| August . | . Normal . | No | No | Yes | Yes | No | No | No | No |
| | Tumour-bearing . | Yes | Yes | Yes | Yes | Yes | Yes | Yes | Yes |
| Hybrid . | . Normal . | No | No | No | No | No | No | No | No |
| | Tumour-bearing . | No | No | No | No | No | No | No | No |

D = Haemolysis in donor's cells.
R = Haemolysis in recipient's cells.

studies are summarized in Table III, from which the following observations may be made :

1. Cells from tumour-bearing hybrid donors do not induce a haemolytic episode in a normal August recipient.
2. Cells from a tumour-bearing August donor do not induce a haemolytic episode in a normal hybrid recipient.
3. Cells from a normal hybrid donor are destroyed to the same extent as cells of the host when transfused to a tumour-bearing August recipient.

Thus it appears that cells of hybrid rats are susceptible to lysis by the haemolytic agent. Consequently, the most probable explanation for the observations that implantation of the tumour in a hybrid host fails to induce a haemolytic episode is that the haemolytic agent is unable to maintain itself and to multiply in the hybrid host.

(*b*) *Type of tumour*.—The effect on red cell survival of the implantation of two other tumours of the August strain has been tested. These are the mammary adenocarcinoma D266 and the melanoma R428. The survival of red cells of rats implanted with the tumour R428 was found to be indistinguishable from normal. However, August rats implanted with the tumour D266 were observed to develop a haemolytic episode 12–14 days after implantation similar to that observed after the implantation of R2426 (Fig. 2). The haemolytic episode induced by the tumour D266 was less acute than that induced by R2426, some 40 per cent of the circulating red cells surviving the episode.

It has been shown (Belcher and Simpson, 1960) that after a haemolytic episode has been induced in a recipient animal by transfusion of blood from a donor bearing the tumour R2426, it was impossible to induce a second episode in the recipient by further injection of blood or transplantation of the tumour. This effect was used in order to test whether the anaemia provoked by implantation of the tumour D266 was produced by a similar agent (Fig. 3). Blood taken from a donor bearing the D266 tumour was transfused to a pair of normal recipients. Fourteen days after transfusion, these rats experienced a mild haemolytic episode. When their haemoglobin levels had returned to normal, the recipients were again transfused, this time with blood from a donor bearing the R2426 tumour. Blood from this donor was also transfused to two normal control animals. Six days later

FIG. 2.—Blood haemoglobin levels and survival of $^{51}$Cr-labelled red cells in two " August " rats implanted with tumour D266.

FIG. 3.—Blood haemoglobin levels in " August " rats transfused with blood from donor rat bearing the tumour R2426.

● ○ } Control recipients.

× + } Recipients previously transfused with blood from donor rat bearing tumour D266.

these controls experienced an acute haemolytic episode. The rats which previously had been transfused with blood from a donor bearing the D266 tumour, however, did not experience a haemolytic episode during the period of observation up to 3 weeks after transfusion. It appears that blood from a donor bearing the tumour D266 is able, subsequently, to "protect" the recipient against the haemolytic episode induced by blood from a R2426 donor. It may be inferred that the agents producing the haemolytic episodes following the implantation of the tumours R2426 and D266 have the same mode of action. It is perhaps significant that both tumours are adenocarcinomata of mammary origin.

*Attentuation of the factor producing haemolytic disease in tumour-bearing rats*

Towards the end of these studies a change occurred in the haemolytic response of August rats following implantation of the tumour R2426. In the last series of rats implanted, the haemolytic episode was not observed until 20–21 days after implantation, instead of after 10–15 days as previously found. As far as could be judged, all other features of the haemolytic disease were unaltered. This development suggests that the haemolytic agent had either become attenuated by numerous passages through the host or that it had undergone some change in its mode of action.

*Production of antibodies to the haemolytic agent*

In experiments designed to detect the presence of circulating antibodies against the haemolytic agent, serum collected from tumour-bearing rats which had recovered from their haemolytic episodes was inactivated by heating to 56° C. Dilutions of tumour extract were incubated with equal volumes of this serum for 1 hour at 37° C. The amount of active haemolytic agent remaining was then estimated by injecting 0·5 ml. amounts of the mixture into pairs of recipient rats. The results of two studies of this kind are summarized in Table IV. In the first two experiments, the treated tumour extracts were injected alone. In the third, they were adsorbed on to normal red cells, in the manner previously described,

TABLE IV.—*Delay in Onset of Haemolytic Episode in Rats Receiving Injections of Tumour Extracts Treated with Antisera*

| Experiment No. | Material injected | Mean interval between injection and onset of haemolytic episode |
|---|---|---|
| 1 | 0·25 ml. tumour extract | 12 days |
| | 0·5 ml. tumour extract + antiserum | 14 days |
| | 1 : 10 dilution tumour extract | 15 days |
| | 1 : 10 dilution tumour extract + antiserum | 20 days |
| 2 | 0·25 ml. tumour extract | 8 days |
| | 0·5 ml. tumour extract + antiserum | 8 days |
| 3 | Red cells treated with tumour extract | 4 days |
| | Red cells treated with 1 : 10 dilution tumour extract + normal serum | 8 days |
| | Red cells treated with 1 : 10 dilution tumour extract + anti-serum | No episode |
| | Red cells treated with 1 : 100 dilution tumour extract + normal serum | 15 days |
| | Red cells treated with 1 : 100 dilution tumour extract + anti-serum | No episode |

before injection. From the results in Table IV, it appears that the haemolytic agent contained in the tumour extract may be partly or wholly inactivated by prior incubation with the antiserum. These findings suggest that circulating antibodies against the haemolytic agent are present, at least during the later stages of tumour growth. However, since it is necessary to dilute the tumour extract by a factor of 10 or 100 to produce a clear demonstration of neutralization, it is likely that the titre of antibody in the serum is low.

### DISCUSSION

Some of the features of the anaemia induced by implantation of the tumour R2426 suggest that it might be attributable to a mechanism similar to that which induces acquired haemolytic anaemias of the auto-immune type (Belcher, 1958, 1959). However, later findings (Belcher and Simpson, 1960), and the results described in the present paper are incompatible with this explanation. There are now good grounds for believing that the agent inducing the haemolytic episode in rate bearing this tumour is infectious and multiplies within the host. The chief evidence for this lies in the observations that the agent responsible for the anaemia may be transferred to the recipient in a minute quantity of blood, that its concentration in the circulating blood of the host increases from the time of implantation and that it may be passaged through a series of hosts without apparent decline in virulence. Further investigations of the properties of the haemolytic agent reveal that it is not filterable, is heat labile and is stable in ether, all findings which suggest that the agent may be a virus (Mackie and McCartney, 1953). Several other workers have described the multiplication of virus in tumour tissue without aetiological relationship to it. Taylor and MacDowall (1949), Law and Dunn (1951), Riley, Lilley, Heurto and Bardell (1960) and Haas (1960) have all reported virus of various pathogenicity, found in mouse tumours, which may be transplanted along with the tumour.

If the conclusion that the virus-like agent is involved in the production of the haemolytic episode is accepted, three possible modes of action of the agent may be postulated.

1. The virus could alter the red cell surface in such a way that the cell became susceptible to haemagglutination, haemolysis or premature destruction in the reticulo-endothelial system.
2. Virus adsorbed to the red cell surface could bind antiviral antibodies rendering the cell susceptible to destruction by any of the above mechanisms.
3. Combination of the virus with the cell surface could render it antigenic. As a result, auto-antibodies could be produced against it.

It is not possible to state categorically which of these explanations is correct, but there are some indications of which is the most probable. Although the results of the neutralization tests suggest that antiviral antibodies are developed, it is improbable that the haemolytic episode is related to their development. The shortest time between a transfusion of blood and the onset of the haemolytic episode is two days and the longest 14 days. It is unlikely that antibody production would come into play in as short a time as 2 days, nor would the rate of production of antibody to the virus be dependent on the amount of virus originally injected. The same objections hold against the hypothesis that the virus changes the red cell surface in such a way that it becomes antigenic. The suggestion that the haemolytic anaemia may develop as a direct result of the virus is a novel one and may not be substantiated on the basis of the present results. It should be noted, however, that a similar mechanism has been postulated as being responsible for the genesis of several cases of haemolytic anaemia of unknown cause, in patients from whose blood several strains of haemagglutinating virus, notably Newcastle Disease Virus, have been isolated (Moolten and Clark, 1952; Moolten, Clark, Glasser, Katz and Miller, 1953).

In the light of the findings described in this paper and a previous communication (Belcher and Simpson, 1960), it is possible to describe the development of acute haemolytic anaemia in rats bearing the R2426 tumour in the following terms. The R2426 tumour is the site of multiplication of an organism with virus-like properties. Following implantation of the tumour into August rats, this organism is released into the circulation and becomes attached to circulating red cells. A point is reached when the number of particles attached to each cell becomes sufficient to cause widespread lysis or agglutination as a result of which there is a sharp fall in blood haemoglobin. Recovery from the haemolytic episode occurs partly as a result of compensating erythropoesis and partly because the circulating red cells become desensitized to the action of the haemolytic agent. The latter effect may possibly be due to blocking of the red cell surface by antiviral antibodies or to the operation of a mechanism which destroys the virus receptor sites on the cell surface. Antibodies to the virus are demonstrable in serum after the haemolytic episode and these may act to keep the virus at the low level. If the tumour is transplanted or blood from a tumour-bearing rat is transfused to a normal recipient, the virus is free of constraint, able to multiply again, and in due course to provoke a haemolytic episode in the recipient.

### SUMMARY

Further investigations of the acute haemolytic anaemia developing in rats of the " August " strain bearing the transplantable tumour R2426 are described. A haemolytic episode could be induced in normal " August " rats by the transfusion of a minute quantity of blood from a recipient bearing the tumour R2426 and the haemolytic agent could be passaged in this way through a series of hosts without apparent deline in virulence. These and other observations suggest that the responsible agent may be a viral infection associated in some way with the tumour. The agent could be demonstrated in a number of tissues of infected " August " rats but failed to maintain itself in the tissues of rats of two strains. The same or a similar agent was shown to be associated with a second transplantable tumour of the " August " strain, the tumour D266. Circulating antibodies to the agent could be demonstrated in serum of rats bearing the tumour R2426 following their haemolytic episode. The haemolytic action of the agent is discussed in the light of these findings.

This work has been supported by a grant from the British Empire Cancer Campaign.

### REFERENCES

BELCHER, E. H.—(1958) 3rd International Symposium on Radioactive Isotopes in Clinical Medicine and Research, BadGastein 1958 (Urban and Schwarzenberg, Munich) p. 206.—(1959) *Acta Un. int. Cancr.*, **15**, 866.

*Idem* AND SIMPSON, S. M.—(1960) *Brit. J. Cancer*, **14**, 224.
HAAS, V.—(1960) *J. nat. Cancer Inst.*, **25**, 75.
LAW, L. W. AND DUNN, T. B.—(1951) *Ibid.*, **11**, 1037.
MACKIE, T. J. AND MCCARTNEY, J. E.—(1953) ' Handbook of Practical Bacteriology '. Edinburgh and London (Livingstone).
MOOLTEN, S. E. AND CLARK, E.—(1952) *Arch. int. Med.*, **89**, 270.
*Iidem*, GLASSER, B. F., KATZ, E. AND MILLER, B. F.—(1953) *Amer. J. Med.*, **14**, 294.
RILEY, V., LILLEY, F., HUERTO, E. AND BARDELL, D.—(1960) *Science*, **132**, 545.
TAYLOR, M. J. AND MACDOWALL, E. C.—(1949) *Cancer Res.*, **2**, 223.